# Systolic‐dominant coronary flow in rats and mice: Challenging the diastolic paradigm across conscious and anaesthetized states

**DOI:** 10.1113/EP092514

**Published:** 2025-09-14

**Authors:** Heidi L. Lujan, Theodore W. Kurtz, Stephen E. DiCarlo

**Affiliations:** ^1^ Department of Physiology College of Osteopathic Medicine, Michigan State University East Lansing Michigan USA; ^2^ Department of Laboratory Medicine University of California San Francisco San Francisco California USA

**Keywords:** cardiac output, diastole, left ventricular pressure, systole

## Abstract

Extensive research in humans, dogs, rabbits, rats, mice and other mammals has consistently demonstrated that coronary blood flow (CBF) peaks during ventricular diastole. For example, studies using transthoracic Doppler echocardiography in anaesthetized rats and mice, isolated blood‐perfused rat hearts and Doppler probes sutured to the myocardial surface have reported diastolic‐dominant CBF. In contrast, while evaluating the effects of dietary salt on coronary vascular resistance in rats, we unexpectedly observed that left CBF peaked during ventricular systole. This observation prompted two follow‐up protocols to test the hypothesis that left coronary flow in rats and mice peaks during systole. In Protocol 1, chronically instrumented conscious male Sprague–Dawley rats were implanted with telemetry pressure sensors and pulsed Doppler flow probes around the ascending aorta and left main coronary artery. Coronary and aortic flow waveforms exhibited nearly identical timing, indicating that CBF peaked during systole. In Protocol 2, anaesthetized, open‐chest, mechanically ventilated rats and mice (both sexes) were studied. Doppler probes and ECG electrodes were used to compare the time from the R wave to the peak of both aortic and coronary flow. Student's paired *t*‐test showed no significant difference between the two, confirming that coronary and aortic flow occur synchronously during systole. These findings demonstrate that, in rats and mice, left coronary blood flow peaks during ventricular systole – not diastole – challenging the widely accepted paradigm. This may reflect structural and haemodynamic features unique to small mammals, such as low ventricular wall tension and high heart rates.

## Abstract (highlights)

- **What is the central question of this study?**
  Given that rats and mice have high heart rates, low ventricular wall tension and rapid systolic blood delivery, does this create an environment where left coronary flow peaks during ventricular systole?
- **What is the main finding and its importance?**
  Extensive research has demonstrated that coronary flow peaks during ventricular diastole. In contrast to the universally accepted view, left coronary blood flow peaked during ventricular systole in rats and mice. Specifically, ascending aortic flow and coronary flow waveforms had nearly identical timing. We offer new insights into coronary haemodynamics in small mammalian species.

## 1 INTRODUCTION

Coronary blood flow, the vital process supplying oxygen and nutrients to the myocardium, is reported to exhibit a well‐established pattern across the cardiac cycle. Specifically, the long‐standing and universally accepted view holds that in humans, as well as in other mammalian species, coronary blood flow through the left coronary artery peaks during ventricular diastole. The proposed mechanisms mediating this phenomenon are multifaceted and are linked to increased vascular resistance mediated by the physical compression of coronary vessels during systolic contraction.

The study of coronary blood flow distribution during the cardiac cycle has garnered substantial attention in many mammalian species. Multiple human studies, largely based on various imaging techniques and catheters with Doppler flow probes, consistently report that at least 70–80% of left coronary blood flow occurs during ventricular diastole (Cole & Hartley, 1977; Davies et al., 2006; Kajiya et al., 1993; Marcus et al., 1999; Seligman et al., 2022). Similarly, studies in dogs have demonstrated a predominance of diastolic coronary blood flow (Green et al., 1935; Vatner et al., 1984). Although, the understanding of coronary flow dynamics in small animals is less well‐defined, left coronary blood flow in rabbits, rats and mice has also been reported to peak during ventricular diastole (Gan et al., 2013; Jones et al., 1994; Sunyecz et al., 2018; Wangler et al., 1981). In rats, investigators using multiple techniques including transthoracic Doppler echocardiography (Gan et al., 2013), direct measurement of flow in isolated blood‐perfused hearts (Bouma et al., 1993) and Doppler ultrasonic flow probes sutured on the surface of the myocardium (Jones et al., 1994) have consistently documented that left coronary blood flow peaks during ventricular diastole.

In contrast, while testing the effects of different salt intakes on coronary vascular resistance in conscious rats, we unexpectedly noted that virtually all left coronary blood flow measured with pulsed Doppler ultrasonic flow probes around the left main coronary artery appears to occur during the systolic phase of the cardiac cycle. Here we report the results of separate studies in which we confirm, in both rats and mice, that coronary blood flow peaks in systole not in diastole. This surprising finding challenges the widely held view of diastolic predominance of left coronary flow in the rats and mice. By reporting these unexpected results and our experimental protocols, we hope to offer new insights into coronary haemodynamics and to enhance the methodological toolkit for studying coronary blood flow in small mammalian species.

In the ensuing sections, we present our methods and results and discuss the implications of our study in the context of existing literature, contributing to the nuanced understanding of a fundamental aspect of cardiovascular physiology.

## 2 METHODS

### 2.1 Animals and ethical approval

Experimental procedures and protocols were reviewed and approved by the Institutional Animal Care and Use Committee at Michigan State University (PROTO202200434) and complied with the National Institutes of Health *Guide for the Care and Use of Laboratory Animals*. Experimental procedures and protocols also complied with *Experimental Physiology*’s policies regarding animal experiments. The investigators understand the ethical principles under which the journal operates and our work complies with the animal ethics checklist.

Twenty‐one male Sprague–Dawley rats, 9–10 weeks of age, were obtained from Inotiv (Indianapolis, IN) and given tap distilled water ad libitum and fed a pelleted purified AIN‐76A diet containing 0.26% NaCl and 0.36% potassium (diet No.100000 from Dyets, Inc., Bethelem, PA, USA). Rats were housed separately in standard rat cages (∼13,350 cm^3^) within a dedicated animal room maintained on 12 h:12 h light–dark cycles where ambient temperature was maintained at the thermoneutral zone for rats (29 ± 1.0°C, 40–60% relative humidity). Each rat was provided a blue, red or amber Rat Retreat (K3170, K3245, and K3246 respectively, Bio‐Serv, Flemington, NJ, USA), Wood Gnawing Blocks (K3515, Bio‐Serv) and The Andersons Bed‐r'Nest (4 g, Animal Specialities and Provisions, Quakertown, PA, USA) for environmental enrichment. Entry into the animal room was restricted to the investigators, ensuring that the animals were not unnecessarily disturbed.

Ten A/J mice (five male and five female; body weight 20–25 g) were obtained from The Jackson Laboratory (A/J Stock no. 000646, Bar Harbor, ME). Mice were housed in Optimice Cages (Animal Care Systems, Centennial, CO, USA) with cotton and paper nestlets (NES3600, Ancare, Bellmore, NY, USA), for environmental enrichment. Mice had free access to chow (Teklad Irradiated Global 19% Protein Extruded Rodent Diet, 2919, Inotiv, Lafayette, IN, USA) and water (tap distilled) and were maintained on 12 h:12 h light–dark cycles, under ambient environmental conditions of 19–24°C and 40–60% relative humidity.

At the end of the experiments, rodents were humanely euthanized. Rodents were deeply anaesthetized with isoflurane followed by removal of the heart.

### 2.2 Experimental protocols

The original plan was to assess coronary blood flow and coronary vascular resistance before and after administration of a high salt diet. However, while measuring coronary blood flow on the control normal salt diet, we unexpectedly observed that coronary flow peaked during ventricular systole rather than in diastole. As a result of the unexpected observation, we designed two complementary protocols to rigorously test the hypotheses that left coronary flow peaks during systole and the majority of flow occurs during systole.

Protocol 1 involved chronically instrumented, intact conscious male rats to assess coronary flow dynamics under physiological conditions, free from the confounding effects of anaesthesia or surgical trauma. To ensure that the observed systolic‐dominant coronary flow was not an artifact of chronically implanted flow probes, Protocol 2 employed acute, open‐chest preparations in anaesthetized, mechanically ventilated rats. This approach enabled direct probe placement over the ascending aorta and left main coronary artery without chronic instrumentation. Protocol 2 was expanded to include both male and female mice, allowing us to examine whether systolic‐dominant coronary flow extended to another small mammalian species and to begin examining potential sex‐based differences. Furthermore, in these acute preparations, surface ECG recordings were obtained, enabling precise timing of aortic and coronary flow waveforms relative to the R wave, thus providing an additional, independent method for evaluating synchrony of aortic and coronary flow during the cardiac cycle.

### 2.3 Protocol 1 chronically instrumented intact conscious rats

#### 2.3.1 Surgical protocol

**FIGURE 1:**
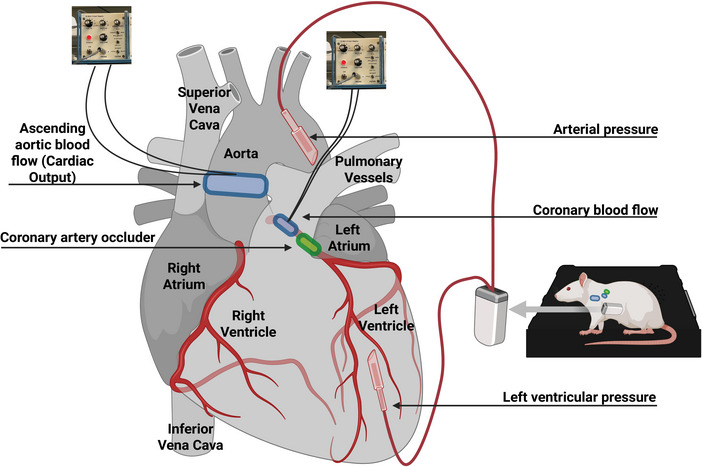
Instrumentation for simultaneous measurement of coronary flow, aortic flow and haemodynamic parameters in the conscious rat. Animals were instrumented with Doppler ultrasonic flow probes placed around the ascending aorta (to measure cardiac output) and the left main coronary artery (to measure coronary blood flow). A coronary artery occluder was also positioned around the left main coronary artery to allow transient occlusion. In addition, telemetry pressure sensors were implanted in the left ventricular cavity and in the descending aorta to continuously record left ventricular and arterial pressure. Note that the Doppler flow probes were positioned at nearly equal distances from the heart, and all flow signals were recorded using the same hardware system to ensure precise temporal synchronization.

Surgery to implant sensors for monitoring arterial blood pressure, left ventricular pressure, left main coronary artery blood flow and ascending aortic blood flow (cardiac output) was performed in male rats with a mean body weight of 316 g (Figure 1).

Animals were anaesthetized initially with 3–5% isoflurane and intubated. Subsequently, animals were supported by a ventilator delivering isoflurane at 1–3%. The disappearance of the pedal (foot) withdrawal reflex was used as an indicator that the animal reached a level of anaesthesia deep enough for the surgical procedures.

Before surgery, animals received atropine (0.05 mg/kg). In addition, multimodal pre‐emptive analgesia was achieved using a local subcutaneous infiltration of bupivacaine (diluted to 0.25%, 5 mg/kg) and lidocaine (diluted to 0.5%, 5 mg/kg) around the incision site and intraperitoneal administration of a non‐steroidal anti‐inflammatory agent (carprofen; 10 mg/kg).

#### 2.3.2 Surgical procedures

Using aseptic conditions and after anaesthesia, radiotelemetry transmitters (HD‐S10 (*n* = 6), Data Sciences, Minneapolis, MN, USA) with attached catheters with pressure‐sensing tips were implanted for arterial pressure monitoring. Specifically, a catheter from a telemetry device was inserted into the left carotid artery and advanced into the descending aorta for recording arterial pressure and heart rate.

Subsequently, the heart was approached via a left thoracotomy through the second intercostal space. In three of these animals, a catheter from an additional radiotelemetry transmitter (HD‐S10‐F2) (*n* = 3) was implanted into the left ventricle to measure left ventricular pressure. The telemetry devices were implanted in the peritoneal cavity and a catheter was placed in the intraperitoneal space for the infusion of fluids and drugs. The catheter was exteriorized on the dorsal aspect of the neck.

To measure ascending aortic flow (cardiac output) in these three animals, the sleeve of the pericardium that extends onto the ascending aorta was dissected free of surrounding tissue (*n* = 3) and an appropriately sized (range 2.5–3.0‐mm) silicone‐type 20 MHz Doppler ultrasonic flow probe (Iowa Doppler Products, Iowa City, IA, USA) was placed around the ascending aorta as previously described in rats and mice (Kulics et al., 1999; Kurtz et al., 2014, 2023; Llewellyn‐Smith et al., 2007; Lujan & DiCarlo, 2013, 2014a). In all six animals, a 1.0 mm silicone‐type 20 MHz Doppler ultrasonic flow probe was positioned around the left main coronary artery to measure coronary blood flow. The flow probe wires were channelled subcutaneously and exited at the dorsal aspect of the neck.

Finally, in the three animals instrumented for simultaneous measurements of left ventricular pressure, cardiac output and coronary flow, a coronary artery occluder, made from 5.0‐gauge atraumatic Ethicon prolene suture, which passed through a polyethlene‐10 guide tubing, was passed around the left main coronary artery 2–3 mm from the origin by inserting the needle into the left ventricular wall under the overhanging left atrial appendage and bringing it out high on the pulmonary conus (*n* = 3). The guide tubing with the other end of the occluder was then exteriorized at the back of the neck. The tubing was filled with a sterile mixture of Vasoline and mineral oil to prevent a pneumothorax. We have used this coronary artery occluder in conscious rats (Collins & Dicarlo, 2005; Lujan & DiCarlo, 2007; Lujan et al., 2006, 2007a, 2007b) and mice (Lujan & DiCarlo, 2014b, 2017).

Note that left main coronary flow was measured in all six rats. However, left ventricular pressure and aortic flow were recorded in a subset of three rats using additional telemetry and Doppler instrumentation. These measurements were added after the initial observation that coronary flow peaked during systole, to further evaluate the timing relationship between ventricular contraction, aortic ejection and coronary perfusion. The reduced sample size for these variables reflects the staged nature of the study and our efforts to minimize procedural complexity and surgical burden.

#### 2.3.3 Post‐operative care in recovery

All animals remained on the feedback‐based temperature control system and ventilator until recovery from the anaesthesia. Once the animals regained consciousness, they were placed in a ThermoCare patented Portable Animal Intensive Care Warmer Unit (DW‐1, Braintree Scientific, Braintree, MA, USA) with a non‐particulate absorbent pad. The animal was monitored and returned to the housing room when fully recovered from the anaesthesia and gained the ability to maintain an upright body position and body temperature. During the recovery period, the rats were provided supplemental enrichment treats (Bio‐Serv, Flemington, NJ, USA), the 0.26% NaCl diet and tap water, and were periodically handled and weighed. Carprofen and buprenorphine (0.1 mg/kg) were administered two times/day for 3 days postoperatively. In addition, the antibiotic cefazolin (10 mg/kg) was administered two times/day for 3 days postoperatively. All peri‐ and postoperative agents were purchased from (Covetrus North America, Columbus, OH).

Rats were allowed to recover for a minimum of 7 days following surgery before experimentation. During this period, animals were monitored daily to ensure stable body weight, thermoregulation and normal behaviour, confirming full physiological recovery before data collection.

#### 2.3.4 Experimental procedure

On the day of the experiment, rats were brought into the laboratory and studied in their standard home cage. Temperature within the cage was maintained within the thermoneutral zone for rats. Beat‐by‐beat, steady‐state haemodynamic variables were continuously recorded for approximately 1 h to ensure stable haemodynamic conditions. To assure our methods could measure coronary blood flow, the left main coronary artery was temporarily occluded by use of the prolene suture. Specifically, acute coronary artery occlusion was performed by pulling up on the suture that was around the left main coronary artery (Collins & Dicarlo, 2005; Lujan & DiCarlo, 2007; Lujan & DiCarlo, 2014b, 2017; Lujan et al., 2006, 2007a, 2007b). A rapid reduction in coronary artery and ascending aortic blood flow and a reduction in arterial pressure occur within seconds of pulling on the suture, documenting coronary artery occlusion. The occlusion was maintained for 30 s. At the end of the experiment, the animals were returned to their housing facilities. On an alternate day, the heart was excised under deep anaesthesia, quickly rinsed clean and weighed. Subsequently, the right ventricular free wall was removed, and the heart reweighed.

#### 2.3.5 Data recording and analysis

A telemetry system was used to record arterial pressure, left ventricular pressure and heart rate data (Data Sciences Inc., St Paul, MN, USA). To record Doppler signals for determining aortic and coronary blood flow, the Doppler wires from the animals were connected via extension wires to ultrasonic, range‐gated pulsed Doppler flow meters with pulse repetition frequencies of 62.5 kHz (Hartley & Cole, 1974).

All signals were continuously sampled at 1 kHz using an analogue‐to‐digital converter (PowerLab 8/35, ADInstruments, Inc., Colorado Springs, CO, USA) and analysed using LabChart Pro software (ADInstruments). PowerLab employs a single analogue‐to‐digital (A/D) converter that samples each channel sequentially within each sampling interval. This results in a small, consistent delay between adjacent channels – approximately 0.25 ‐ seconds (250 ms) in our set‐up. For figure preparation, we corrected this timing misalignment between channels by using the ‘Shift’ function in LabChart. Waveform data were then exported as text files and re‐plotted using external graphing software to allow for standardized formatting and alignment.

#### 2.3.6 Calculations

The pulsed Doppler method was used to determine aortic blood flow (cardiac output) and left main coronary flow as follows. Blood flow ($\dot{Q}$), in mL/min, was calculated by:

$$\dot{Q}=\mathrm{kHz}\;\mathrm{of}\;\mathrm{Doppler}\;\mathrm{frequency}\;\mathrm{shift}\times K.$$

where *K* = 1.24 × *d* ^2^, (where *d* is the cuff diameter of the flow probe). Using this system, the Doppler shift frequency (kHz) is directly proportional to blood flow.

Total peripheral resistance (systemic vascular resistance) was calculated by:

$$\begin{array}{ccc} & & \mathrm{Total}\;\mathrm{peripheral}\;\mathrm{resistance}\;\left(\mathrm{mmHg}/\mathrm{mL}/\min\right)\\ & & \;=\;\mathrm{mean}\;\mathrm{arterial}\;\mathrm{pressure}\;\left(\mathrm{mmHg}\right)/\mathrm{cardiac}\;\mathrm{output}\;\left(\mathrm{mL}/\min\right) \end{array}$$

Coronary vascular resistance was calculated by:

$$\begin{array}{ccc} & & \mathrm{Coronary}\;\mathrm{vascular}\;\mathrm{resistance}\;\left(\mathrm{mmHg}/\mathrm{mL}/\min\right)\\ & & \;=\;\mathrm{mean}\;\mathrm{arterial}\;\mathrm{pressure}\;\left(\mathrm{mmHg}\right)/\mathrm{coronary}\;\mathrm{blood}\;\mathrm{flow}\;\left(\mathrm{mL}/\min\right) \end{array}$$

### 2.4 Protocol 2, anaesthetized, open chest, mechanically ventilated rats and mice

To independently test our unexpected findings and to assure that placement of the chronically implanted pulsed Doppler flow probes did not alter coronary blood flow dynamics, we studied anaesthetized, open chest, mechanically ventilated rats. We also studied mice to determine whether the systolic‐dominant coronary flow pattern observed in rats extends to another commonly used small mammalian species with a similarly low ventricular radius‐to‐wall thickness ratio. We studied both male and female mice to evaluate whether sex‐related differences in left ventricular geometry or coronary vessel size alter the mechanical conditions that permit coronary flow during systole. Including both sexes ensures that our findings are not limited by sex‐specific anatomical variation and improves the generalizability of the systolic flow phenomenon in small mammals.

#### 2.4.1 Surgical procedures

Animals were anaesthetized initially with 3–5% isoflurane and intubated. Subsequently, animals were supported by a ventilator delivering isoflurane at 1–3%. The disappearance of the pedal (foot) withdrawal reflex was used an indicator that the animal reached a level of anaesthesia deep enough for the surgical procedures. Before surgery, animals received intraperitoneal administration of a nonsteroidal anti‐inflammatory agent (carprofen; 10 mg/kg).

#### 2.4.2 Experimental procedures

**FIGURE 2:**
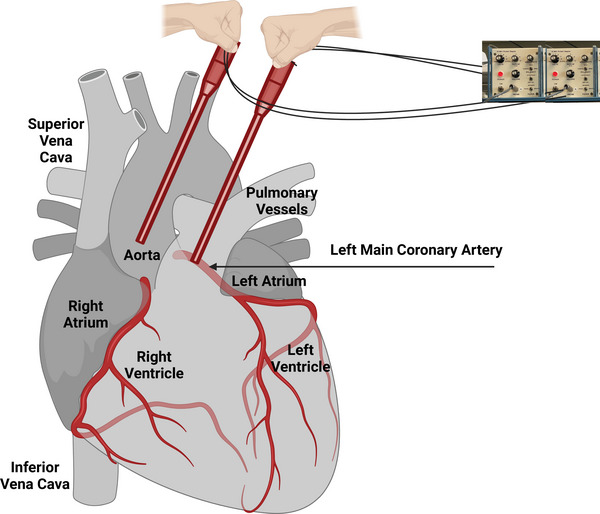
Simultaneous Doppler recordings of ascending aortic and coronary flow velocities in anaesthetized, mechanically ventilated rats and mice. A thoracotomy was performed in anaesthetized and mechanically ventilated rats and mice. 20 MHz pulsed Doppler tubing probes were held directly over the ascending aorta and left main coronary artery. This procedure enabled simultaneous recording of ascending aortic and left main coronary artery blood flow velocities.

A total of 15 rats were studied under acute, open‐chest, mechanically ventilated conditions. In the first cohort, eight male rats were anaesthetized as described above, and the ascending aorta and left main coronary artery were exposed via thoracotomy. Twenty megahertz pulsed Doppler tubing probes (part number 385‐0002‐01, Indus Instruments, Webster, TX, USA) were held directly over the ascending aorta and left main coronary artery to record blood flow velocity waveforms (Figure 2). In this setting, the Doppler crystal was embedded flat at the tip of a pencil‐style probe and placed flush against the vessel. This orientation produces an angle of insonation approximately 90 degrees, meaning the ultrasound beam is directed perpendicular to the direction of blood flow. While this angle is suboptimal for precise velocity quantification due to the minimal Doppler shift at 90° (cos 90° = 0), it is sufficient and reliable for assessing waveform timing, which was the primary objective of these measurements.

Specifically, although the insonation angle was near 90°, slight deviations from perpendicular – common in practical applications – allowed for the detection of usable Doppler frequency shifts. These signals provided clear resolution of flow onset, peak and reversal, enabling detection of both forward and reverse flow components. As a result, the timing relationship between aortic and coronary flow waveforms could be accurately assessed despite the suboptimal angle for flow magnitude estimation.

In a subsequent cohort, seven additional male rats were studied under the same open‐chest preparation, but with the addition of surface ECG electrodes placed to record the R wave. This allowed for precise timing of peak flow velocities relative to electrical cardiac events.

To assess whether the pattern of coronary flow observed in rats extended to another small mammalian species, and to begin examining potential sex‐based differences, 10 mice (five males and five females) were also studied under similar acute, open‐chest conditions. Doppler probes were positioned over the ascending aorta and left main coronary artery, and surface ECG electrodes were placed to enable synchronization of flow and electrical recordings. These data allowed for both cross‐species comparison and initial exploration of sex‐related differences in coronary flow timing.

## 3 RESULTS

### 3.1 Chronically instrumented intact conscious rats

**FIGURE 3:**
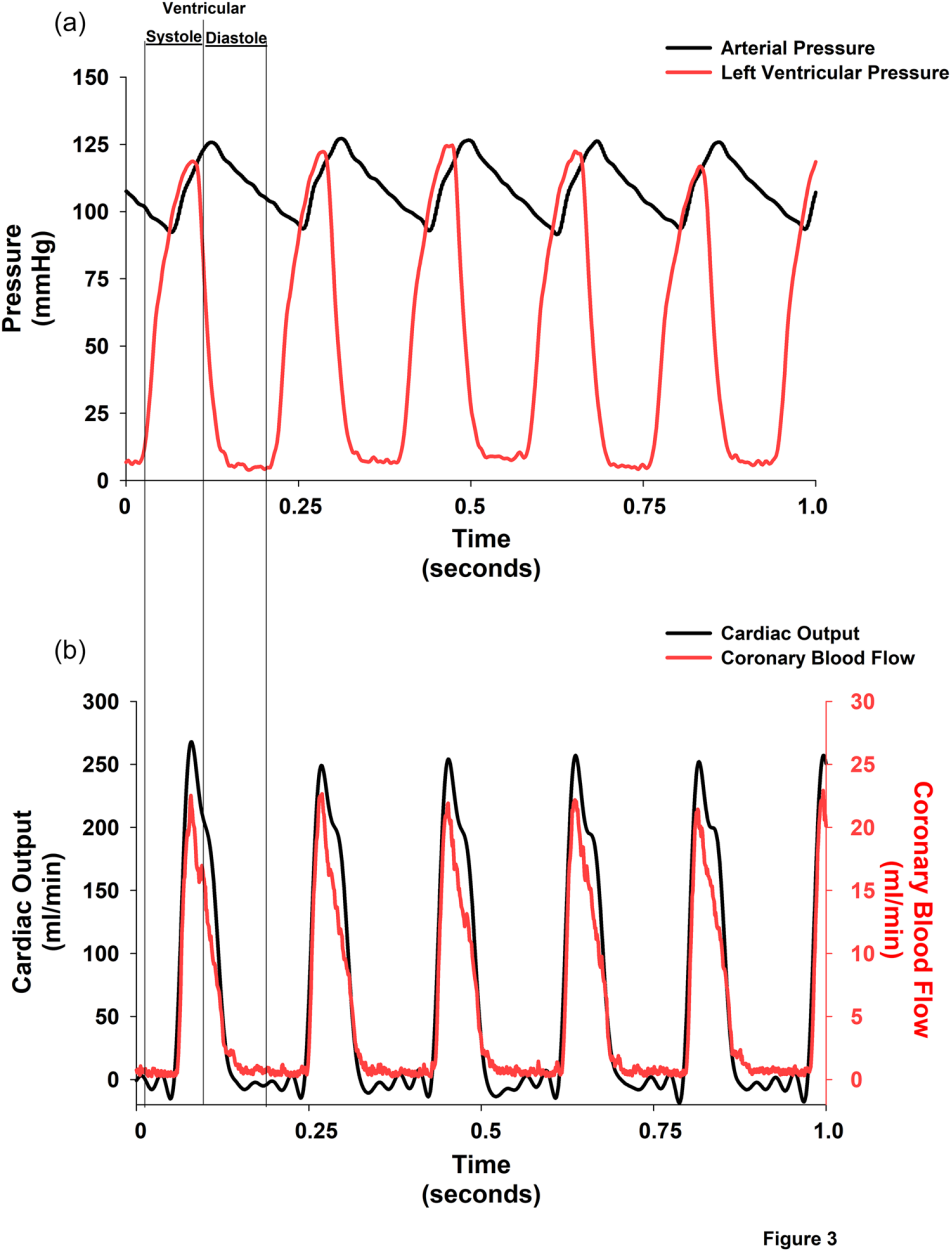
Simultaneous haemodynamic and doppler flow recordings in a conscious, chronically instrumented rat. Recording of arterial pressure and left ventricular pressure (a), and ascending aortic and left coronary blood flow (b) in an intact, conscious rat. In panel (b), separate *y*‐axes are used: the *y*‐axis for cardiac output (ascending aortic flow) is on the left, and the *y*‐axis for left coronary artery flow is on the right. This format accommodates the large difference in flow magnitude between the two vessels. Importantly, the timing of the aortic and coronary flow waveforms was nearly identical, demonstrating that left coronary blood flow peaked during ventricular systole. Vertical reference lines denote the systolic and diastolic phases of one cardiac cycle.

Figure 3 shows an original recording of the signals for arterial pressure, left ventricular pressure (Figure 3a), aortic (cardiac output) and left coronary blood flow (Figure 3b) in an intact conscious rat. Note that Figure 3b presents separate *y*‐axes for cardiac output and coronary blood flow with different scales. The *y*‐axis for cardiac output is on the left side of the figure. The *y*‐axis for coronary blood flow is on the right side of the figure. We used this format because ascending aortic flow was much larger than left coronary artery blood flow. Importantly, the aortic flow and left main coronary flow waveforms had nearly identical timing, documenting that left coronary blood flow peaked during ventricular systole. Both waveforms showed downward deflections with isovolumic ventricular contraction and both showed upward waveforms during rapid ventricular ejection. In addition, the coronary blood flow signal occurred during the systolic phase of the ventricular pressure waveform.

**FIGURE 4:**
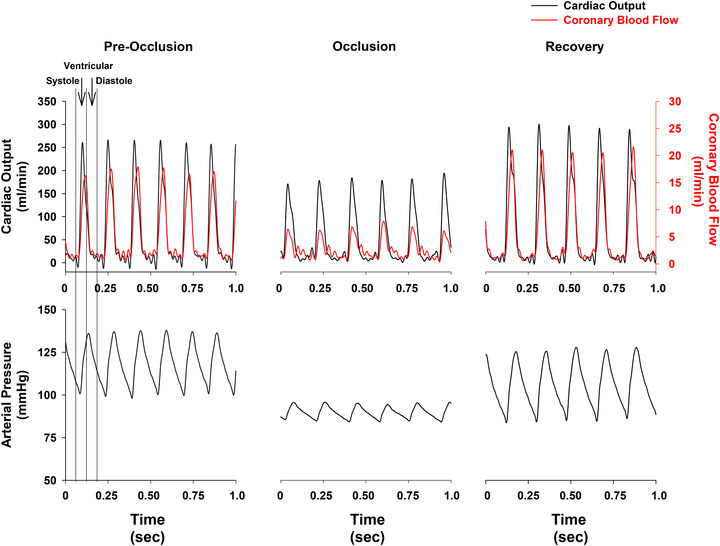
Effect of coronary occlusion on cardiac output and coronary blood flow in a conscious rat. Recording of cardiac output (ascending aortic flow), left coronary artery blood flow, and arterial pressure during left main coronary artery occlusion in a chronically instrumented, conscious rat. Separate *y*‐axes are used for cardiac output and coronary flow: the *y*‐axis for cardiac output is shown on the left, and the *y*‐axis for coronary flow is on the right. This dual‐axis format was necessary due to the larger magnitude of aortic flow relative to coronary flow. Vertical reference lines demarcate the systolic and diastolic phases of a representative cardiac cycle. Occlusion of the left main coronary artery markedly reduced coronary blood flow, yet the temporal relationship between aortic and coronary flow was preserved. This finding reinforces that left coronary perfusion occurs almost entirely during ventricular systole, and that the systolic dominance of coronary flow persists even when flow is experimentally reduced.

Figure 4 is an analogue recording showing that occlusion of the left main coronary artery in a conscious rat reduced coronary blood flow but did not alter the relationship between aortic blood flow and left coronary blood flow documenting that virtually all left coronary blood flow occurred during the systolic phase of the cardiac cycle. Again, note that separate *y*‐axes for cardiac output and coronary blood flow are shown with different scales. The *y*‐axis for cardiac output is on the left side of the figure. The *y*‐axis for coronary blood flow is on the right side of the figure. We used this format because ascending aortic flow was much larger than left coronary artery blood flow.

Table 1 presents means and 95% confidence intervals (CI) for resting arterial blood pressure, heart rate, left ventricular systolic and diastolic pressures, cardiac output (without left coronary blood flow because the piezoelectric sensor was distal to the sinuses of Valsalva), left main coronary artery blood flow, left main coronary blood flow/gram of left ventricle, left main coronary blood flow as percentage of cardiac output, systemic vascular resistance and coronary vascular resistance in the intact conscious rat. Note that coronary blood flow to the left ventricle averaged 6.0 (95% CI, 5.74–6.3) mL/min/g of left ventricular weight and approximately 7.5% (95% CI, 5.34–9.6%) of cardiac output) in conscious rats. These values are consistent with reports using radioactively labelled microspheres (Miller et al., 1980). Specifically, Miller and colleagues reported that coronary blood flow to the entire heart in un‐anaesthetized control rats averaged 7.6 mL/min/g which was 6.6% of cardiac output. These values are significantly higher than values reported for man. Specifically, under baseline‐resting conditions, coronary blood flow to the left ventricle averages ∼0.5–1.0 mL/min/g in man (Tune, 2014) and is approximately 5% of cardiac output (Deussen et al., 2012).

**TABLE 1:** Haemodynamic parameters and coronary flow characteristics in conscious rats.

Haemodynamic parameter	Value; mean (95% CI)
Mean arterial pressure (mmHg)	108.7 (103.5–113.9) (*n* = 6)
Heart rate (bpm)	342.7 (303.9–381.5) (*n* = 6)
Left ventricular systolic pressure (mmHg)	147.1 (111.1–183.0) (*n* = 3)
Left ventricular diastolic pressure (mmHg)	3.7 (0.56–6.8) (*n* = 3)
Cardiac output (mL/min)	71.4 (45.8–97.0) (*n* = 3)
Left main coronary blood flow (mL/min)	5.5 (4.9–6.2) (*n* = 6)
Left main coronary blood flow per gram of left ventricular tissue (mL/min/g)	6.0 (5.7–6.3) (*n* = 6)
Left main coronary blood flow as percentage of cardiac output (%)	7.5 (5.3–9.6) (*n* = 3)
Systemic vascular resistance (mmHg/mL/min)	1.6 (0.9–2.2) (*n* = 3)
Coronary vascular resistance (mmHg/mL/min)	19.9 (17.1–22.7) (*n* = 6)

Resting arterial pressure, heart rate, left ventricular systolic and diastolic pressures, cardiac output (excluding left coronary flow due to probe placement distal to the sinuses of Valsalva), left main coronary artery blood flow, coronary flow normalized to left ventricular mass, coronary flow as a percentage of cardiac output, systemic vascular resistance, and coronary vascular resistance measured in intact, conscious rats.

### 3.2 ECG‐anchored timing of aortic and coronary flow

**FIGURE 5:**
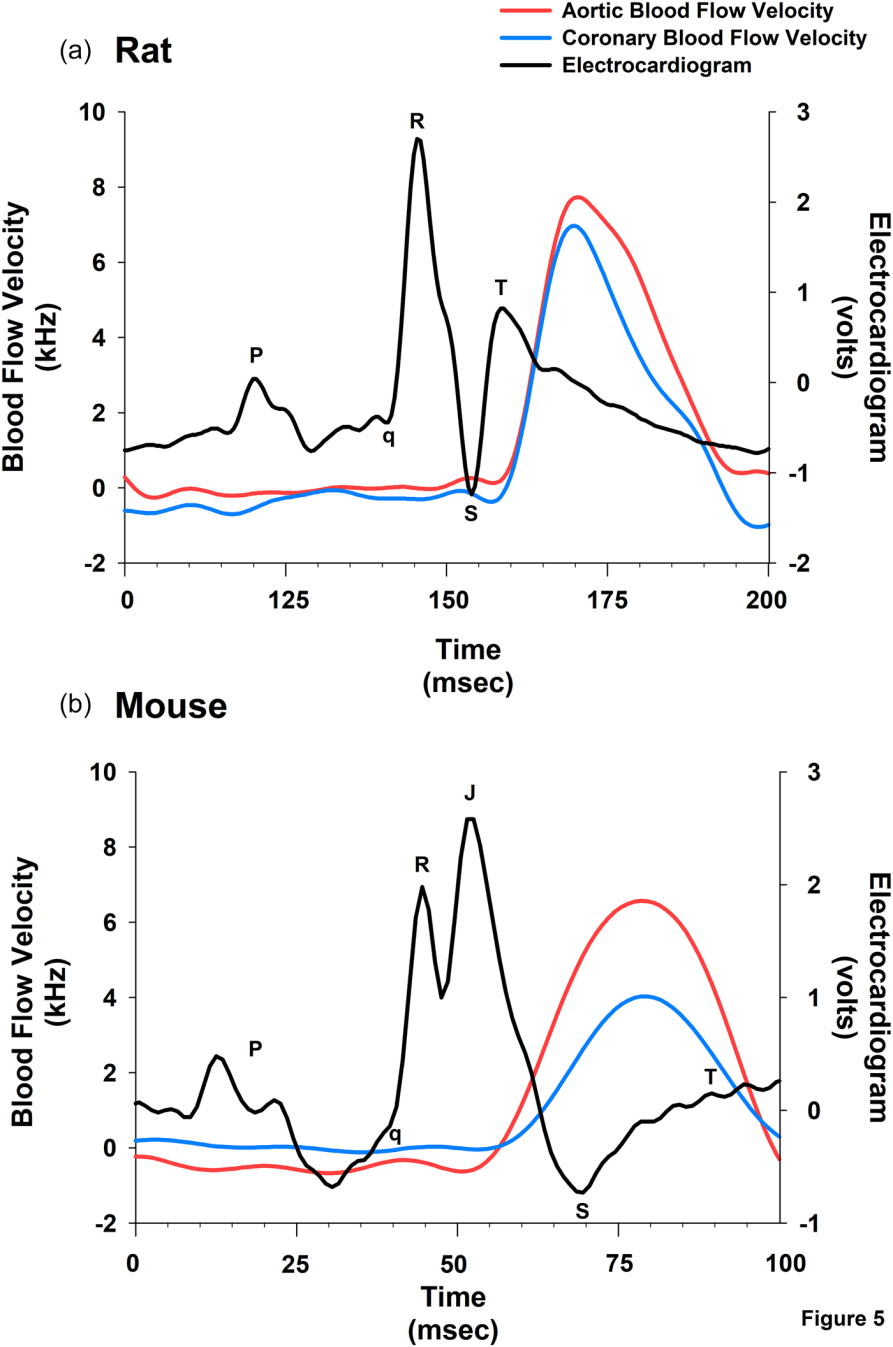
Representative synchronized ECG, aortic flow, and coronary flow waveforms from anaesthetized, open‐chest rats and mice. Tracings were recorded in seven male rats, five male mice, and five female mice using pulsed Doppler probes placed over the ascending aorta and left main coronary artery, along with surface ECG electrodes. All signals were acquired simultaneously using synchronized hardware to ensure precise timing alignment across channels. Each panel displays one complete cardiac cycle, defined by the interval between successive P waves. In panel (a) (rat), the ECG trace spans approximately 200 ms and includes a full P–QRS–T complex followed by the onset of the next P wave, consistent with a heart rate of ∼300 bpm. In panel (b) (mouse), the trace spans approximately 100 ms and similarly captures a full P–QRS–T cycle, matching a heart rate of ∼600 bpm. ECG wave components (P wave, QRS complex, T wave) have been labelled in both panels to aid interpretation. Note that in the mouse the ST segment is not isoelectric and has a characteristic J‐wave that represents early repolarization. The timing of peak coronary and aortic flow velocity relative to the R wave was determined from these synchronized recordings. Additionally, to assess mechanical synchrony independent of electrical activation, the time interval between the peak of ascending aortic flow (designated as time zero) and peak coronary flow velocity was calculated within each cardiac cycle.

**FIGURE 6:**
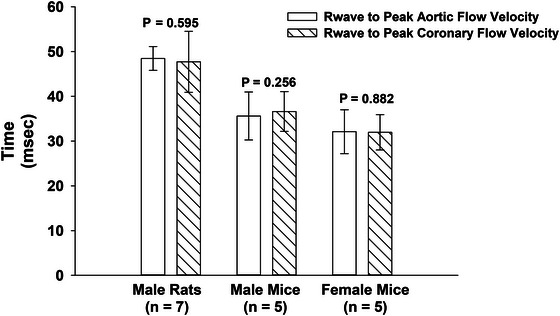
ECG‐anchored timing of aortic and coronary flow peaks in rats and mice. The time (mean and 95% CI) from the R wave of the ECG to the peak of ascending aortic and left coronary artery flow was measured in anaesthetized male rats (*n* = 7), male mice (*n* = 5), and female mice (*n* = 5). Each animal served as its own control. Paired comparisons showed no significant difference between the timing of aortic and coronary flow peaks across all groups (*P* > 0.25), supporting that left coronary flow peaks during systole in synchrony with aortic ejection.

To further assess the timing relationship between peak aortic and coronary flow, we measured the interval from the R wave of the ECG to the peak of each flow signal within the same cardiac cycle (Figure 5). This analysis was conducted in anaesthetized animals equipped with surface ECG electrodes, including male rats (*n* = 7), male mice (*n* = 5), and female mice (*n* = 5). Across all groups, Student's paired *t*‐test revealed no significant differences between the R wave‐to‐peak intervals for aortic and coronary flow (all *P* > 0.25), indicating close temporal synchrony of peak aortic flow and peak coronary flow. As shown in Figure 6, these results confirm that left coronary flow consistently peaks during ventricular systole, in tight alignment with aortic ejection, regardless of species or sex (Figures 5 and 6).

While we recognize the value of ECG‐anchored analysis, we caution that aligning mechanical flow events (Doppler waveforms) to the electrical R wave can be physiologically misleading. The R wave reflects ventricular depolarization, which precedes mechanical contraction and ejection by a variable delay due to excitation–contraction coupling, vascular compliance and haemodynamic inertia. These delays are not uniform and may introduce interpretive artifacts when comparing across animals or conditions. Thus, we emphasize that direct comparison of mechanical events (i.e. flow‐to‐flow synchrony) provides the most physiologically meaningful interpretation – given the variability introduced by excitation–contraction coupling and differing propagation speeds of electrical and mechanical signals.

### 3.3 Peak‐to‐peak timing between aortic and coronary flow

**FIGURE 7:**
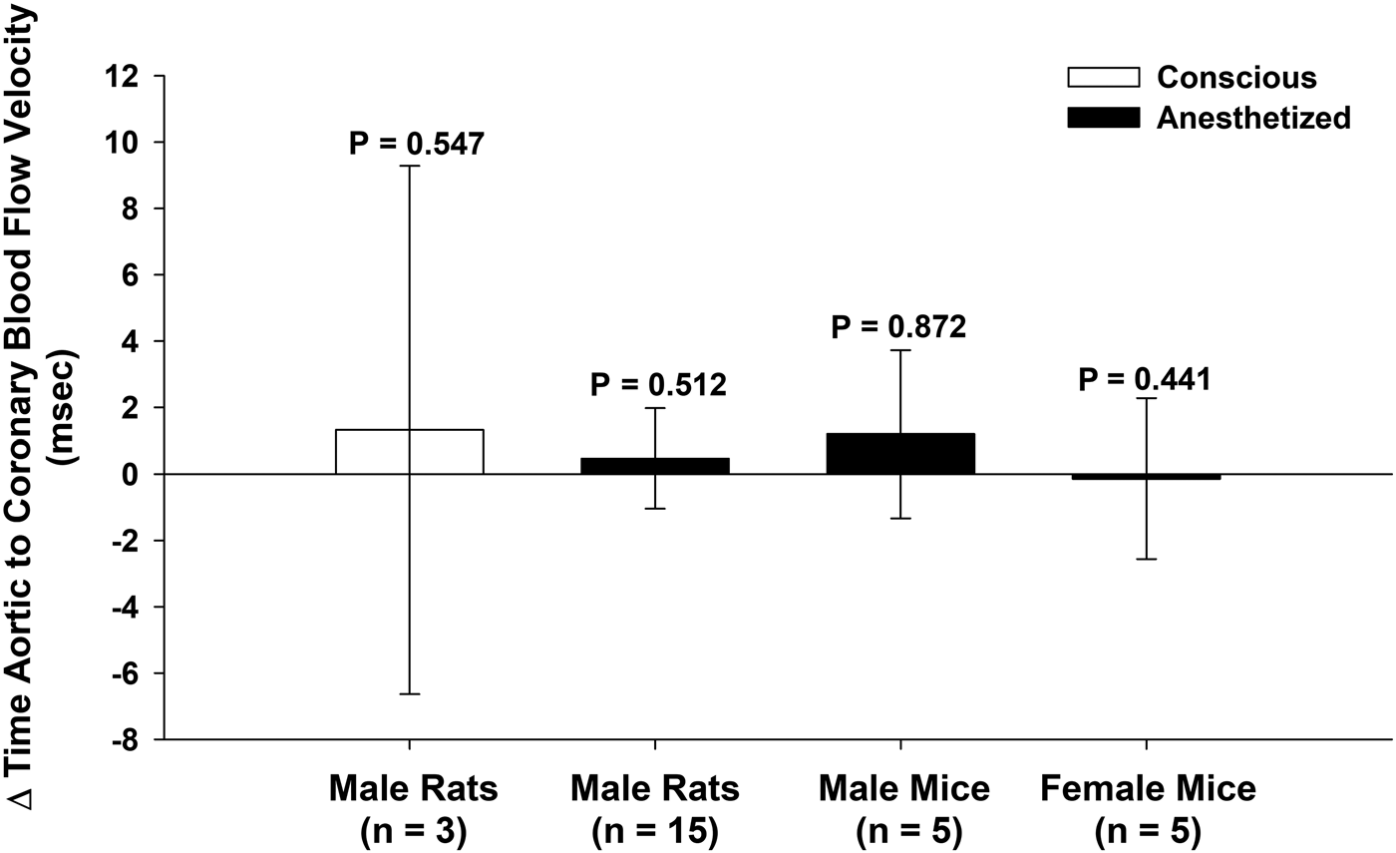
Timing between peak aortic and peak coronary flow in rats and mice. The difference (Δ*t*, mean and 95% CI) between the peak of ascending aortic flow velocity and peak left coronary artery flow velocity for each cardiac cycle, with the aortic peak defined as time zero. This peak‐to‐peak analysis was conducted in conscious male rats (*n* = 3), anaesthetized male rats (*n* = 15), male mice (*n* = 5) and female mice (*n* = 5). In all groups, the mean Δ*t* was small and not significantly different from zero (all *P* > 0.2, one‐sample *t*‐test), indicating that coronary flow peaks synchronously with aortic ejection across species, sexes and physiological states. These findings support the robustness of systolic‐dominant coronary perfusion.

To evaluate the temporal alignment of coronary and aortic blood flow, we also measured the time difference between the peak of ascending aortic flow (designated as time zero) and the peak of left coronary flow within the same cardiac cycle (Figure 6). This analysis was performed in both conscious rats (*n* = 3) and anaesthetized animals, including rats (*n* = 15), male mice (*n* = 5), and female mice (*n* = 5). All flow waveforms were recorded using a single synchronized Doppler system (Figure 7).

Across all groups, the timing difference (Δ*t*) between aortic and coronary peak flow was small and not significantly different from zero (all *P* > 0.2; one‐sample *t*‐test), consistent with the view that coronary flow peaks during systole, in close synchrony with peak aortic flow. These results (Figure 7), indicate that peak coronary flow tightly coincides with peak aortic flow in different rodent species, sexes and physiological states (conscious or anaesthetized).

### 3.4 Absolute coronary volume flow during systole and diastole

**FIGURE 8:**
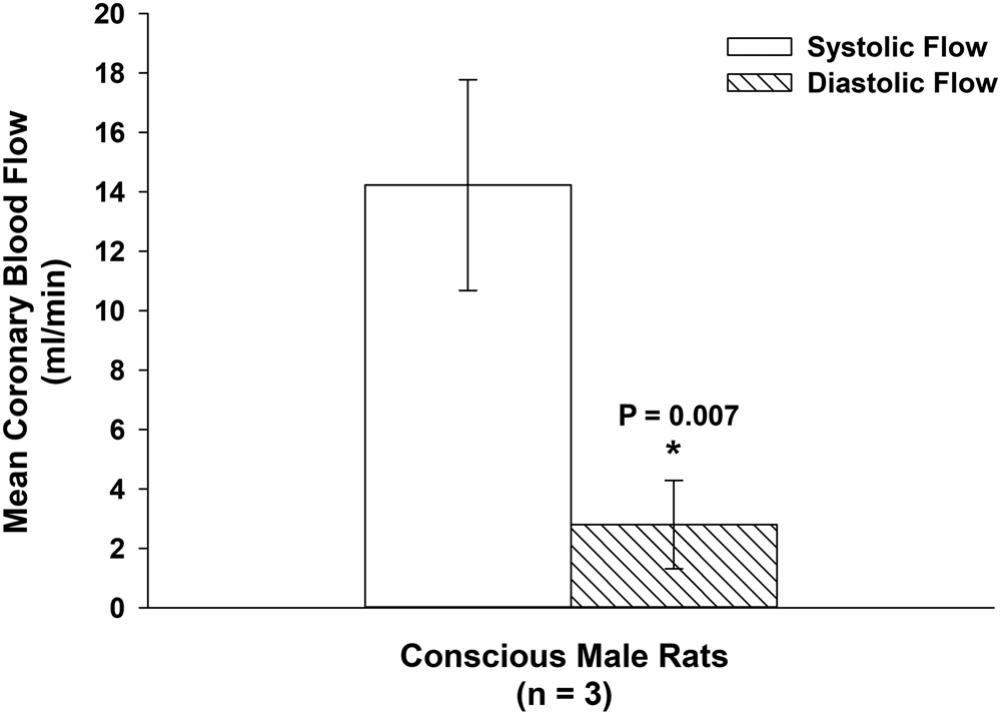
Absolute coronary flow during systole and diastole in conscious rats. Coronary blood flow (mL/min, mean and 95% CI) during ventricular systole and diastole in conscious, chronically instrumented male rats (*n* = 3). Left ventricular pressure recordings were used to define the start and end of each cardiac phase. The Doppler flow waveform was integrated over each phase to calculate absolute volume flow per cycle. Systolic flow was significantly greater than diastolic flow (*P* = 0.0074, paired *t*‐test), supporting a systolic‐dominant coronary perfusion pattern under unanaesthetised physiological conditions.

In addition to testing whether coronary flow and aortic flow peak together during ventricular systole, we determined whether absolute coronary volume flow is greater during ventricular systole than during diastole. In three conscious, chronically instrumented rats, we integrated the Doppler flow waveforms over each phase of the cardiac cycle, with systole and diastole defined by aortic valve opening and closure, identified from simultaneously recorded left ventricular pressures. This enabled us to determine mean systolic and diastolic flow volumes for each animal across each cardiac cycle. As shown in Figure 8, mean systolic coronary flow (14.2 mL/min; 95% CI, 10.7–17.8) was significantly greater than mean diastolic flow (2.8 mL/min; 95% CI, 1.31–4.29; *P* = 0.0074). These findings (Figure 8), demonstrate that in conscious physiological conditions, left coronary flow is not only aligned with ventricular systole but is also systolic‐dominant in magnitude.

### 3.5 Velocity versus volume clarification

Although ascending aortic blood flow is approximately 10‐fold higher than coronary flow (Figure 3), the measured flow velocities were only modestly different (Figure 5). This observation is explained by the relationship: $\dot{Q}$ = *v* × *A*, where flow ($\dot{Q}$) equals velocity (*v*) multiplied by cross‐sectional area (*A*). Although blood velocity in the coronary artery is similar to blood velocity in the aorta, the lower cross‐sectional area of the coronary artery than of the aorta causes coronary volume blood flow to be lower than aortic volume blood flow. These acute experiments (Figure 5) measured velocity only, as the Doppler crystals were applied directly to the vessel wall without measuring or controlling cross sectional area (Figure 5). The peak of the volume flow occurs at the same time as the peak of the velocity.

## 4 DISCUSSION

In contrast to the longstanding consensus that left coronary blood flow peaks during diastole, the present study demonstrates that in both rats and mice, coronary flow consistently peaks during ventricular systole and occurs in tight synchrony with ascending aortic flow. This unexpected pattern was first identified in chronically instrumented, conscious rats and was then rigorously confirmed through two independent timing analyses in anaesthetized, open‐chest animals of both sexes. By leveraging synchronized Doppler recordings and ECG‐based timing, we provide robust evidence that systolic‐dominant coronary perfusion is a reproducible physiological feature of small rodents. These findings challenge the generalizability of diastolic‐predominant coronary flow models and highlight the importance of species‐specific cardiac structure and haemodynamics in shaping coronary perfusion dynamics.

Previous research in humans, dogs, rabbits, rats, mice and other mammals (Bouma et al., 1993; Davies et al., 2006; Gan et al., 2013; Green et al., 1935; Jones et al., 1994; Sunyecz et al., 2018; Vatner et al., 1984) universally documented that left coronary blood flow peaks during the diastolic phase of the cardiac cycle. For example, investigators using multiple techniques including transthoracic Doppler echocardiography in anaesthetized rats (Gan et al., 2013), isolated blood‐perfused rat hearts (Bouma et al., 1993) and Doppler ultrasonic flow probes sutured on the surface of the myocardium of conscious rats (Jones et al., 1994) have consistently reported that left coronary blood flow peaks during diastole. It is believed that this pattern of coronary blood flow during the cardiac cycle underlies the integral role of diastolic relaxation in facilitating optimal coronary perfusion.

Recent studies using transthoracic colour Doppler imaging coupled with ECG in mice (Chang et al., 2021; Guo et al., 2023; Wikström et al., 2005) provide additional context for evaluating coronary flow dynamics in small mammals. These investigations consistently report diastolic‐dominant flow profiles under baseline conditions and have been instrumental in advancing non‐invasive assessments of coronary flow reserve (CFR) and microvascular function. However, these studies primarily focus on evaluating coronary flow in response to ischaemia–reperfusion injury, pressure overload or atherosclerosis rather than determining the precise timing of peak coronary flow within the cardiac cycle. Moreover, transthoracic imaging techniques face inherent limitations in acoustic window alignment, insonation angle and reliance on indirect timing references. In contrast, our study employed a direct mechanical comparison of ascending aortic and left main coronary Doppler waveforms, recorded simultaneously using identical hardware under both conscious and anaesthetized conditions. This approach enables precise assessment of flow‐to‐flow timing, revealing that coronary and aortic flows peak concurrently during ventricular systole. By complementing ECG‐anchored analysis with direct mechanical timing, our findings improve upon prior Doppler studies and provide robust evidence for systolic‐dominant coronary perfusion in rats and mice.

We do not know the reasons for the disparate results between our unexpected findings and all previous reports, but discuss methodological issues that may provide important insights. The transthoracic Doppler echocardiography technique is reportedly prone to user bias (Sunyecz et al., 2018). The guidelines established by the American Society of Echocardiography state that measurements from only three to four cardiac cycles deemed representative by the rater are sufficient to obtain an average, yet multiple studies have shown significant differences between intra‐ and inter‐operator variability in transthoracic Doppler echocardiography analysis (Finegold et al., 2013; Galderisi et al., 1992; Quiñones et al., 2002). Transthoracic Doppler echocardiography can also be laborious and requires restraint of the animals or anaesthesia, both of which are known stressors of the cardiovascular system. Furthermore, the amplitude and intensity of ultrasound waves decrease as they travel through tissue and the transducer angle to the vessel can vary as the investigator explores with the probe. These problems are not encountered with Doppler ultrasonic probes implanted around the coronary artery.

Furthermore, these limitations are less pronounced when placing the Doppler probe directly over the vessel, as in our open‐chest studies. The shorter distance reduces signal loss, and manual stabilization of the probe reduces angle variability. Although the probe was held manually, the signals were robust and reproducible. Crucially, coronary and aortic flows were recorded using the same hardware system, ensuring synchronized data acquisition and eliminating timing offsets due to mismatched systems.

The study reporting peak left coronary blood flow during diastole in isolated blood‐perfused rat hearts (Bouma et al., 1993) may not mimic the clinical or physiological situation. Specifically, isolated perfused heart preparations are devoid of humoral influences and neuronal regulation. Furthermore, adenosine was administered to obtain maximum coronary dilatation and the heart was paced. Specifically, the sinoatrial node was destroyed with formalin and the right ventricle was stimulated with a constant pacing frequency just above the heart's own rhythm. Finally, to obtain static cardiac contractions the calcium channels of the sarcoplasmic reticulum were blocked with ryanodine. Thus, results performed in the isolated blood‐perfused rat heart should be confirmed in complex, conscious systems.

The study reporting peak left coronary blood flow during diastole with the Doppler ultrasonic flow probes sutured on the surface of the myocardium (Jones et al., 1994) raises several methodological issues. It is important to note that the diastolic phase of the cardiac cycle was determined by the arterial pressure waveform. However, the arterial pressure sensor was positioned distal (downstream) to the flow sensor, which created a misalignment of arterial pressure and blood flow waves. Furthermore, aligning the pressure wave, which travels rapidly through the arterial walls, with the blood flow wave, which moves more slowly through the blood vessels is problematic. The pressure wave, moving at speeds of 4–6 m/s, is a mechanical pulse reflecting the elastic properties of the arterial walls and the force of the heart's contraction. In contrast, the blood flow wave, traveling at about 0.5 m/s, represents the actual movement of blood propelled by the heart.

Aligning these waves incorrectly conflates their distinct velocities and physiological roles, leading to inaccurate interpretations of coronary blood flow dynamics, the timing of myocardial perfusion, and the assessment of cardiovascular function. To illustrate this concept, we draw on the analogy of lightning and thunder. While both lightning and thunder originate from the same event (ventricular systole), light (pressure wave) and sound (blood flow) travel at different speeds and are detected at different times.

This misalignment underscores the necessity for precise differentiation between pressure and blood flow waves in cardiovascular research. Investigators should avoid aligning the pressure wave and the blood flow wave as if they were synchronous and traveling at the same speed. We avoided this error by showing that the ascending aortic flow and coronary flow waveforms had nearly identical timing, documenting that left coronary blood flow peaked during ventricular systole.

Unlike previous reports that relied on visual inspection of waveforms, we performed formal statistical comparisons of timing between aortic and coronary peak flow. We employed two approaches. First, in rats and mice of both sexes with ECG, we compared the time from the R wave to the peak of each flow waveform. Second, we directly compared peak aortic and coronary flow timing using aortic flow as the reference (time zero). These analyses consistently revealed no significant delay in coronary flow, strengthening the validity of our observations (Figures 6 and 7).

To rule out anaesthesia‐related artifacts, we also analysed chronically instrumented, conscious rats. Even in these unrestrained animals, peak coronary and aortic flows occurred nearly simultaneously, further supporting our conclusions (Figure 7). In addition, mean systolic and diastolic flow volumes were calculated in conscious animals across cardiac cycles. These findings, presented in Figure 8, demonstrate that in conscious physiological conditions, left coronary flow is not only aligned with systole but is also systolic‐dominant in magnitude.

Our findings also suggest that this systolic‐dominant pattern is not sex‐dependent. In both male and female mice, the timing of coronary and aortic flow peaks was nearly identical. This supports the notion that coronary perfusion timing in small mammals is governed by structural and biomechanical factors rather than sex differences in autonomic tone or myocardial properties.

Our unexpected findings may provide new insights related to the control of coronary blood flow during the cardiac cycle in rats, mice and perhaps other small mammals. It is generally believed that coronary blood flow and ascending aorta blood flow are asynchronous due to strong compressive forces exerted on the coronary vasculature during ventricular systole that increase coronary vascular resistance. The resultant increases in coronary vascular resistance limit or prevent increase in coronary blood flow during systole when aortic flow is increasing. However, based on the Law of Laplace discussed below, we believe that the amount of ventricular tension (compressive force) on the coronary vasculature during systole may be low in rats, mice and in other small mammals. Low ventricular tension (compressive force) would limit the level of coronary resistance achieved during systole and thereby allow for increases in coronary flow that are synchronous with increases in aortic flow.

The Law of Laplace holds that the tension in the wall of a cylindrical membrane is the product of pressure and radius divided by wall thickness (Wall tension = Pressure × Radius/Wall thickness). The level of pressure achieved during systole is similar in rats and in humans. However, because the rat has a small ratio of internal ventricular radius/ventricular wall thickness (1.23 in rats vs. 2.7 in humans; Grossman et al., 1975; Omens et al., 1998), the level of ventricular wall tension/compressive force exerted on the coronary vasculature during systole may not be sufficient to raise coronary resistance to levels that prevent coronary blood flow from increasing during ventricular contraction.

**FIGURE 9:**
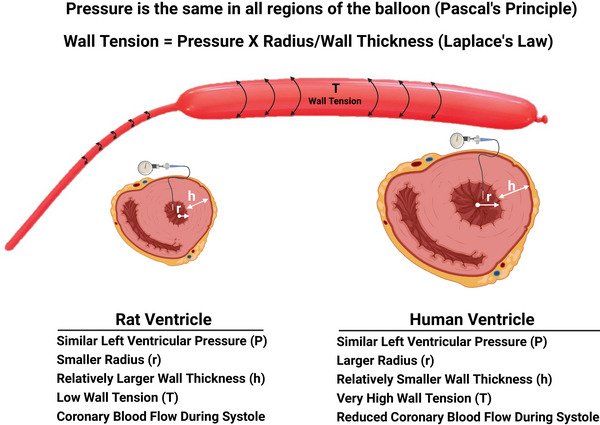
Application of the Law of Laplace to explain systolic coronary flow in small mammals. According to the Law of Laplace, wall tension in a cylindrical structure is proportional to the product of pressure and radius divided by wall thickness (Tension = Pressure × Radius/Wall thickness). Because intraventricular pressure is comparable between rats and humans, differences in wall tension arise primarily from geometric factors. The human ventricle, with a larger internal radius and relatively thinner wall, experiences significantly higher wall tension during systole – sufficient to compress intramyocardial vessels and restrict coronary flow. In contrast, the rat ventricle has a smaller radius and relatively thicker wall, resulting in lower systolic wall tension. This reduced compressive force permits left coronary vessels to remain patent during systole, enabling the observed pattern of systolic‐dominant coronary perfusion in small mammals.

This explanation involving the Law of Laplace can be modelled by considering the forces that develop during inflation of a long cylindrical balloon. When you inflate a long cylindrical balloon, only one part of the balloon initially expands (Figure 9). Continue inflating it and the balloon expands towards the balloon's end. Consider the expanded part of the balloon as a human ventricle with a high ratio of radius/wall thickness (Figure 9). Consider the much less expanded part of the balloon as a rat ventricle with a relatively smaller ratio of radius/wall thickness (Figure 9). Based on Pascal's principle, the pressure inside the balloon is the same throughout. That is, pressure is the same in the expanded and less‐expanded regions of the balloon. However, compressing the expanded, inflated end of the balloon (representing the human ventricle) and the less‐expanded end of the balloon (representing the rat ventricle) illustrates the impact of the radius/wall thickness ratio on wall tension as expressed in Laplace's Law. Specifically, the tension of the inflated part of the balloon with the large ratio of internal radius/wall thickness is considerably greater than the tension of the less‐inflated part of the balloon with the lower ratio of internal radius/wall thickness.

How does high wall tension, if present, compress the coronary vasculature? As the myocardium contracts and wall tension builds, the ventricular wall shortens, thickens and deforms. This active mechanical deformation compresses the intramural coronary vessels – especially in the subendocardium – reducing their radius or even collapsing them transiently. Thus, while wall tension itself is not a direct compressive agent, it reflects the magnitude of stress that ultimately gets transmitted as deformation, leading to vascular compression and increased resistance.

Since vascular resistance is inversely proportional to the fourth power of vessel radius (Poiseuille's Law), even small reductions in coronary diameter from myocardial tension can cause large increases in resistance. Thus, while tension does not directly compress vessels, it reflects the mechanical forces driving deformation that ultimately elevate coronary resistance during systole in large mammals.

It is also important to note that in rats and mice, the heart operates at very high rates (300–600 beats/min), which drastically shortens the duration of each phase of the cardiac cycle, including diastole. With such a brief diastolic period, there is limited opportunity for coronary perfusion to occur during this phase alone. Moreover, the anatomical proximity of the coronary ostia to the aortic root, coupled with the rapid systolic ejection of blood, enables blood to reach the coronary arteries almost immediately. This set‐up, paired with minimal wall tension, allows the rodent heart to fully rely on systolic flow for coronary perfusion, challenging the traditional notion that diastole is the primary phase for coronary blood flow.

In summary, our findings challenge the widely held view that in both small and large mammals, coronary blood flow peaks mainly during diastole and is asynchronous with ascending aortic flow. The current findings in rats and mice show that coronary blood flow peaks during ventricular systole and is synchronous with aortic flow. Thus, in contrast to the conventional view that coronary blood flow primarily peaks during diastole, evidence suggests that all coronary flow in the rat and mouse peaks during ventricular systole. This systolic dominance is largely attributable to markedly smaller wall tension acting on the coronary vessels during ventricular systole in rodents compared to humans.

Taken together, our findings across species, sexes and experimental conditions converge on a consistent physiological principle: in small mammals with high heart rates and low ventricular wall tension, coronary flow occurs predominantly during systole. This observation challenges the long‐standing dogma of diastolic coronary dominance and highlights the importance of species‐specific cardiac geometry in shaping coronary perfusion patterns. These insights may help refine the interpretation of coronary dynamics in small‐animal models and improve translational cardiovascular research. Specifically, a clearer understanding of species‐specific coronary dynamics is essential for accurate extrapolation of preclinical data to human conditions.

## AUTHOR CONTRIBUTIONS

The experiments were performed in the laboratory of Stephen E. DiCarlo, Department of Physiology, Michigan State University, East Lansing, Michigan, USA. Heidi L. Lujan, Theodore W. Kurtz and Stephen E. DiCarlo conceived and designed the research, Heidi L. Lujan, Theodore W. Kurtz and Stephen E. DiCarlo acquired, analysed, or interpreted data for the work, Heidi L. Lujan, Theodore W. Kurtz and Stephen E. DiCarlo, drafted or revised the work critically for important intellectual content. All authors have read and approved the final version of this manuscript and agree to be accountable for all aspects of the work in ensuring that questions related to the accuracy or integrity of any part of the work are appropriately investigated and resolved. All persons designated as authors qualify for authorship, and all those who qualify for authorship are listed.

## CONFLICT OF INTEREST

None declared.

## FUNDING INFORMATION

None.

## Data Availability

The data that support the findings of this study are available from the corresponding author (S.E.D.), upon reasonable request.

## References

[eph70043-bib-0001] Bouma, P. , Sipkema, P. , & Westerhof, N. (1993). Coronary arterial inflow impediment during systole is little affected by capacitive effects. American Journal of Physiology, 264, H715–H721. 8456974 10.1152/ajpheart.1993.264.3.H715

[eph70043-bib-0002] Chang, W. T. , Fisch, S. , Dangwal, S. , Chen, M. , Cheng, S. , Chen, Z. C. , & Liao, R. (2021). Angiotensin II blockers improve cardiac coronary flow under hemodynamic pressure overload. Hypertension Research, 44(7), 803–812. 33568793 10.1038/s41440-021-00617-1 PMC8260442

[eph70043-bib-0003] Cole, J. S. , & Hartley, C. J. (1977). The pulsed Doppler coronary artery catheter preliminary report of a new technique for measuring rapid changes in coronary artery flow velocity in man. Circulation, 56(1), 18–25. 862168 10.1161/01.cir.56.1.18

[eph70043-bib-0004] Collins, H. L. , & DiCarlo, S. E. (2005). Acute exercise increases the ventricular arrhythmia threshold via the intrinsic adenosine receptor system in conscious hypertensive rats. American Journal of Physiology‐Heart and Circulatory Physiology, 289(3), H1020–H1026. 15879488 10.1152/ajpheart.00156.2005

[eph70043-bib-0005] Davies, J. E. , Whinnett, Z. I. , Francis, D. P. , Manisty, C. H. , Aguado‐Sierra, J. , Willson, K. , Foale, R. A. , Malik, I. S. , Hughes, A. D. , Parker, K. H. , & Mayet, J. (2006). Evidence of a dominant backward‐propagating “suction” wave responsible for diastolic coronary filling in humans, attenuated in left ventricular hypertrophy. Circulation, 113(14), 1768–1778. 16585389 10.1161/CIRCULATIONAHA.105.603050

[eph70043-bib-0006] Deussen, A. , Ohanyan, V. , Jannasch, A. , Yin, L. , & Chilian, W. (2012). Mechanisms of metabolic coronary flow regulation. Journal of Molecular and Cellular Cardiology, 52(4), 794–801. 22004900 10.1016/j.yjmcc.2011.10.001

[eph70043-bib-0007] Finegold, J. A. , Manisty, C. H. , Cecaro, F. , Sutaria, N. , Mayet, J. , & Francis, D. P. (2013). Choosing between velocity‐time‐integral ratio and peak velocity ratio for calculation of the dimensionless index (or aortic valve area) in serial follow‐up of aortic stenosis. International Journal of Cardiology, 167(4), 1524–1531. 22575631 10.1016/j.ijcard.2012.04.105

[eph70043-bib-0008] Galderisi, M. , Benjamin, E. J. , Evans, J. C. , D'Agostino, R. B. , Fuller, D. L. , Lehman, B. , Wolf, P. A. , & Levy, D. (1992). Intra‐ and interobserver reproducibility of Doppler‐assessed indexes of left ventricular diastolic function in a population‐based study (the Framingham Heart Study). American Journal of Cardiology, 70(15), 1341–1346. 1442588 10.1016/0002-9149(92)90772-q

[eph70043-bib-0009] Gan, L. M. , Wikström, J. , & Fritsche‐Danielson, R. (2013). Coronary flow reserve from mouse to man–from mechanistic understanding to future interventions. Journal of Cardiovascular Translational Research, 6(5), 715–728. 23877202 10.1007/s12265-013-9497-5 PMC3790920

[eph70043-bib-0010] Green, H. D. , Gregg, D. D. , & Wiggers, C. J. (1935). The phasic changes in coronary flow established by differential pressure curves. American Journal of Physiology, 112(4), 627–639.

[eph70043-bib-0011] Grossman, W. , Jones, D. , & McLaurin, L. P. (1975). Wall stress and patterns of hypertrophy in the human left ventricle. Journal of Clinical Investigation, 56(1), 56–64. 124746 10.1172/JCI108079 PMC436555

[eph70043-bib-0012] Guo, Z. , Wang, A. , Gao, Y. , Xie, E. , Ye, Z. , Li, Y. , Zhao, X. , Shen, N. , & Zheng, J. (2023). Dynamic Assessments of Coronary Flow Reserve after Myocardial Ischemia Reperfusion in Mice. Journal of Visualized Experiments, Aug 25(198). 10.3791/65391 37677023

[eph70043-bib-0013] Hartley, C. J. , & Cole, J. S. (1974). An ultrasonic pulsed Doppler system for measuring blood flow in small vessels. Journal of Applied Physiology, 37(4), 626–629. 4411990 10.1152/jappl.1974.37.4.626

[eph70043-bib-0014] Jones, L. F. , Landas, S. K. , & Johnson, A. K. (1994). Measurement of coronary blood flow velocity in conscious rats. American Journal of Physiology, 266, H840–H845. 8141384 10.1152/ajpheart.1994.266.2.H840

[eph70043-bib-0015] Kajiya, F. , Matsuoka, S. , Ogasawara, Y. , Hiramatsu, O. , Kanazawa, S. , Wada, Y. , Tadaoka, S. , Tsujioka, K. , Fujiwara, T. , & Zamir, M. (1993). Velocity profiles and phasic flow patterns in the non‐stenotic human left anterior descending coronary artery during cardiac surgery. Cardiovascular Research, 27(5), 845–850. 8348583 10.1093/cvr/27.5.845

[eph70043-bib-0016] Kulics, J. M. , Collins, H. L. , & DiCarlo, S. E. (1999). Postexercise hypotension is mediated by reductions in sympathetic nerve activity. American Journal of Physiology‐Heart and Circulatory Physiology, 276(1), H27–H32. 10.1152/ajpheart.1999.276.1.H27 9887013

[eph70043-bib-0017] Kurtz, T. W. , Lujan, H. L. , & DiCarlo, S. E. (2014). The 24 h pattern of arterial pressure in mice is determined mainly by heart rate‐driven variation in cardiac output. Physiological Reports, 2(11), e12223. 25428952 10.14814/phy2.12223 PMC4255824

[eph70043-bib-0018] Kurtz, T. W. , jr Morris, R. C. , Pravenec, M. , Lujan, H. L. , & DiCarlo, S. E. (2023). Hypertension in primary aldosteronism is initiated by salt‐induced increases in vascular resistance with reductions in cardiac output. Hypertension, 80(5), 1077–1091. 37043613 10.1161/HYPERTENSIONAHA.123.20953

[eph70043-bib-0019] Llewellyn‐Smith, I. , Martin, C. , Fenwick, N. , DiCarlo, S. , Lujan, H. , & Schreihofer, A. (2007). VGLUT1 and VGLUT2 innvervation in autonomic regions of intact and transected rat spinal cord. Journal of Comparative Neurology, 503(6), 741–767. 17570127 10.1002/cne.21414

[eph70043-bib-0020] Lujan, H. L. , Britton, S. L. , Koch, L. G. , & DiCarlo, S. E. (2006). Reduced susceptibility to ventricular tachyarrhythmias in rats selectively bred for high aerobic capacity. American Journal of Physiology‐Heart and Circulatory Physiology, 291(6), H2933–H2941. 16891405 10.1152/ajpheart.00514.2006

[eph70043-bib-0021] Lujan, H. L. , & DiCarlo, S. E. (2007). T5 spinal cord transection increases susceptibility to reperfusion‐induced ventricular tachycardia by enhancing sympathetic activity in conscious rats. American Journal of Physiology‐Heart and Circulatory Physiology, 293(6), H3333–H3339. 17933964 10.1152/ajpheart.01019.2007

[eph70043-bib-0022] Lujan, H. L. , & DiCarlo, S. E. (2013). Cardiac output, at rest and during exercise, before and during myocardial ischemia, reperfusion, and infarction in conscious mice. American Journal of Physiology‐Regulatory, Integrative and Comparative Physiology, 304(4), R286–R295. 23302959 10.1152/ajpregu.00517.2012 PMC3567356

[eph70043-bib-0023] Lujan, H. L. , & DiCarlo, S. E. (2014a). Cardiac electrophysiology and the susceptibility to sustained ventricular tachycardia in intact, conscious mice. American Journal of Physiology‐Heart and Circulatory Physiology, 306(8), H1213–H1221. 24561859 10.1152/ajpheart.00780.2013 PMC3989750

[eph70043-bib-0024] Lujan, H. L. , & DiCarlo, S. E. (2014b). Reperfusion‐induced sustained ventricular tachycardia, leading to ventricular fibrillation, in chronically instrumented, intact, conscious mice. Physiological Reports, 2(6), e12057. 24973331 10.14814/phy2.12057 PMC4208649

[eph70043-bib-0025] Lujan, H. L. , & DiCarlo, S. E. (2017). Fundamental hemodynamic mechanisms mediating the response to myocardial ischemia in conscious paraplegic mice: Cardiac output versus peripheral resistance. Physiological Reports, 5(6), e13214. 10.14814/phy2.13214 PMC5371571 28336819

[eph70043-bib-0026] Lujan, H. L. , Kramer, V. A. , & DiCarlo, S. E. (2007a). Electro‐acupuncture decreases the susceptibility to ventricular tachycardia in conscious rats by reducing cardiac metabolic demand. American Journal of Physiology‐Heart and Circulatory Physiology, 292(5), H2550–H2555. 17209007 10.1152/ajpheart.00979.2006

[eph70043-bib-0027] Lujan, H. L. , Kramer, V. J. , & DiCarlo, S. E. (2007b). Sex Influences the susceptibility to reperfusion‐induced sustained ventricular tachycardia and beta‐adrenergic receptor blockade in conscious rats. American Journal of Physiology‐Heart and Circulatory Physiology, 293(5), H2799–H2808. 17630345 10.1152/ajpheart.00596.2007

[eph70043-bib-0028] Marcus, J. T. , Smeenk, H. G. , Kuijer, J. P. , Van der Geest, R. J. , Heethaar, R. M. , & Van Rossum, A. C. (1999). Flow profiles in the left anterior descending and the right coronary artery assessed by MR velocity quantification: Effects of through‐plane and in‐plane motion of the heart. Journal of Computer Assisted Tomography, 23(4), 567–576. 10433289 10.1097/00004728-199907000-00017

[eph70043-bib-0029] jr Miller, E. D. , Kistner, J. R. , & Epstein, R. M. (1980). Whole‐body distribution of radioactively labelled microspheres in the rat during anesthesia with halothane, enflurane, or ketamine. Anesthesiology, 52(4), 296–302. 7362048 10.1097/00000542-198004000-00002

[eph70043-bib-0030] Omens, J. H. , Vaplon, S. M. , Fazeli, B. , & McCulloch, A. D. (1998). Left ventricular geometric remodeling and residual stress in the rat heart. Journal of Biomechanical Engineering, 120(6), 715–719. 10412454 10.1115/1.2834884

[eph70043-bib-0031] Quiñones, M. A. , Otto, C. M. , Stoddard, M. , Waggoner, A. , & Zoghbi, W. A. (2002). Recommendations for quantification of Doppler echocardiography: A report from the Doppler quantification task force of the nomenclature and standards committee of the American Society of Echocardiography. Journal of the American Society of Echocardiography, 15(2), 167–184. 11836492 10.1067/mje.2002.120202

[eph70043-bib-0032] Seligman, H. , Nijjer, S. S. , van de Hoef, T. P. , de Waard, G. A. , Mejía‐Rentería, H. , Echavarria‐Pinto, M. , Shun‐Shin, M. J. , Howard, J. P. , Cook, C. M. , Warisawa, T. , Ahmad, Y. , Androshchuk, V. , Rajkumar, C. , Nowbar, A. , Kelshiker, M. A. , van Lavieren, M. A. , Meuwissen, M. , Danad, I. , Knaapen, P. , …, Petraco, R. (2022). Phasic flow patterns of right versus left coronary arteries in patients undergoing clinical physiological assessment. EuroIntervention, 17(15), 1260–1270. 34338643 10.4244/EIJ-D-21-00189 PMC9724998

[eph70043-bib-0033] Sunyecz, I. L. , McCallinhart, P. E. , Patel, K. U. , McDermott, M. R. , & Trask, A. J. (2018). Defining coronary flow patterns: Comprehensive automation of transthoracic doppler coronary blood flow. Scientific Reports, 8(1), 17268. 30467422 10.1038/s41598-018-35572-4 PMC6250694

[eph70043-bib-0034] Tune, J. D. (2014). Coronary circulation. In Colloquium series on intergrated systems physiology: From molecule to function to disease. 6(3), 1–189.

[eph70043-bib-0035] Vatner, S. F. , Pasipoularides, A. , & Mirsky, I. (1984). Measurement of arterial pressure‐dimension relationships in conscious animals. Annals of Biomedical Engineering, 12(5), 521–534. 6442831 10.1007/BF02363921

[eph70043-bib-0036] Wangler, R. D. , Peters, K. G. , Laughlin, D. E. , Tomanek, R. J. , & Marcus, M. L. (1981). A method for continuously assessing coronary blood flow velocity in the rat. American Journal of Physiology, 241(6), H816–H820. 7325249 10.1152/ajpheart.1981.241.6.H816

[eph70043-bib-0037] Wikström, J. , Grönros, J. , Bergström, G. , & Gan, L. M. (2005). Functional and morphologic imaging of coronary atherosclerosis in living mice using high‐resolution color Doppler echocardiography and ultrasound biomicroscopy. Journal of the American College of Cardiology, 46(4), 720–727. 16098442 10.1016/j.jacc.2005.04.053

